# Assessment of source material for human intestinal organoid culture for research and clinical use

**DOI:** 10.1186/s13104-022-05925-4

**Published:** 2022-02-10

**Authors:** Paulo C. M. Urbano, Hamish C. K. Angus, Safina Gadeock, Michael Schultz, Roslyn A. Kemp

**Affiliations:** 1 Department of Microbiology and Immunology, University of Otago, Dunedin, New Zealand; 2 Department of Medicine, University of Otago, Dunedin, New Zealand; 3 Children’s Hospital Los Angeles, University of Southern California, Los Angeles, USA; 4 Department of Gastroenterology, Southern District Health Board, Dunedin, New Zealand

**Keywords:** Organoids, IBD, Intestine, Inflammation, Development, Epithelium, Cryopreservation

## Abstract

**Objective:**

Human intestinal organoids (hIOs) have potential as a model for investigating intestinal diseases. The hIO system faces logistic challenges including limited access to biopsies or low expression of epithelial cell types. Previous research identified the feasibility of tissue from the transverse (TC) or sigmoid colon (SC), or from cryopreserved biopsies from regions of the gastrointestinal tract. We aimed to create a protocol for robust hIO generation that could be implemented across multiple centres, allowing for development of a consistent biobank of hIOs from diverse patients.

**Results:**

TC and SC hIOs were expanded from fresh or frozen biopsies with standard or refined media. The expression of epithelial cells was evaluated via PCR. Growth of TC and SC hIO from healthy donors was reproducible from freshly acquired and frozen biopsies. A refined media including insulin-like growth factor (IGF)-1 and fibroblast growth factor (FGF)-2 enabled the expression of epithelial cells, including higher expression of goblet cells and enterocytes compared to standard organoid media. We identified a consistent time point where hIOs generated from frozen biopsies reflect similar hIO composition from freshly acquired samples. Feasibility of hIOs as a tool for research and clinical use, including the use of frozen biopsies, was demonstrated.

**Supplementary Information:**

The online version contains supplementary material available at 10.1186/s13104-022-05925-4.

## Introduction

Three dimensional human intestinal organoids (hIOs), derived from patient intestinal stem cells isolated from intestinal crypts, have considerable potential as a tool for investigating human intestinal diseases, such as inflammatory bowel diseases (IBD) [1–12]. Organoids have been used to investigate the function of the human intestinal epithelial barrier [13, 14]. The implementation of hIO technology in research and clinical settings faces many logistical challenges, including the need for sufficient samples from a limited number of patients and the invasiveness of the procedures required to collect biopsies for research purposes. The limitation of samples can also be explained by patient heterogeneity and individual research study criteria. We have combined research findings from three areas—site of biopsy [15], use of refined media [8] and cryopreservation [16], to design an optimised protocol for robust hIO generation under conditions that would facilitate collaborative research across multiple centres.

## Main text

### Methods

Biopsies derived from TC and SC of healthy people (n = 9, Additional file 1: Table S1) undergoing colonoscopy were collected at the Department of Gastroenterology, Southern District Health Board. From each patient, six biopsies (2.8 mm × 8.8 mm) were obtained either from the TC or SC using jumbo-biopsy forceps (Radial Jaw 4 Jumbo Forceps w/ Needle, Boston Scientific, MA, USA).

#### Isolation of intestinal crypts and generation of human intestinal organoids

Intestinal biopsies were collected with the following media: Advanced Dulbecco’s Modified Eagle Medium (DMEM) + F12 (Invitrogen, MA, USA), fungizone (2.5 µg/mL, Invitrogen), penicillin and streptavidin (1%, Thermo Fisher Scientific, MA, USA), fetal calf serum (FCS; 10%, Invitrogen), gentamicin (0.05 mg/mL, Invitrogen), normocin (0.1 mg/mL, Integrated Science, Sydney, Australia). For _frozen_hIOs, fresh biopsies were suspended in Recovery Cell Culture Freezing Medium (Invitrogen) and stored at -80 °C overnight, then transferred to liquid nitrogen and stored for up to 1 month. Both fresh or thawed biopsies were washed with phosphate buffered saline (PBS; Sigma-Aldrich, MS, USA) plus dithiothreitol (DTT;10 mM, Sigma-Aldrich), and suspended in PBS plus ethylenediaminetetraacetic acid (EDTA; 8 mM, BDH, Dubai, UAE) on ice for 60 min. EDTA was removed, and the biopsies were suspended on cold PBS. The biopsies were shaken and supernatant enriched with crypts isolated. Crypts were centrifuged at 40 g at 4 °C for 3 min to remove debris. Intestinal crypts were resuspended in Matrigel® (25 µL of crypts/Matrigel, Corning, NY, USA), pipetted on pre-warmed Nunclon Delta Surface 24 well flat-bottom plates (Thermo Fisher Scientific) and incubated for 15 min at 37 °C and 5% CO_2_ to allow polymerisation of Matrigel. 500 µL of standard media (SM) or refined media (RM) were added to wells and crypts incubated at 37 °C and 5% CO_2_ up to 20 days without passage. Culture media was refreshed every 2–3 days. Organoids derived from frozen biopsies were first incubated up to two days with RM plus Rock inhibitor (0.01 mM, Y-27632, Biogems, CA, USA).

#### Reverse transcription polymerase chain reaction

Organoids were released from Matrigel using Cell Recovery Solution (Corning). Total RNA was extracted using the RNeasy Plus Micro Kit (Qiagen, Hilden, Germany) followed by cDNA synthesis using the SuperScript III First-Strand Synthesis System and Oligo(dT)20 primer (Thermo Fisher Scientific). TaqMan™ Gene Expression Master Mix (Thermo Fisher Scientific), and gene expression assay were used for RT-PCR (Additional file 1: Table S2). The Relative Quantification app (Thermo Fisher Scientific cloud) was used for data analysis. RT-qPCR cycle values (C_T_) obtained for specific mRNA expression in each sample were normalised to C_T_ values of human endogenous (housekeeping) gene *HPRT1* (hypoxanthine phosphoribosyltransferase 1) resulting in ΔC_T_ values (log ratio of the gene concentrations) and used to calculate relative gene expression [17]:

ΔC_T =_ *Mean CT of gene of interest – Mean CT housekeeping gene.*

We performed an exponential conversion of ΔC_T_, namely 2^−ΔCT^, using:

*2^(exponential) – ΔCT*

#### Statistics

Statistical analysis was performed using GraphPad Prism 8.0 (GraphPad Software, California, United States). For experiments with more than two groups of matched samples, ANOVA one-way followed by Sidak’s multiple comparisons was used (95% confidence interval).

### Results and discussion

#### *Sigmoid colon-derived organoids can be generated *ex vivo* and are similar to transverse colon-derived organoids*

HIOs have been generated from the gastrointestinal tract including the stomach [18], small intestine [6], terminal ileum [19], transverse colon (TC), sigmoid colon (SC) [19, 20], and rectum [21]. A colonoscopy to collect TC biopsies is invasive, requires sedation and prolonged recovery time, and is costly. Collection of SC biopsies is less invasive, usually causes less discomfort and is performed without sedation. A flexible sigmoidoscopy is therefore a feasible alternative for research purposes.

**Fig. 1:**
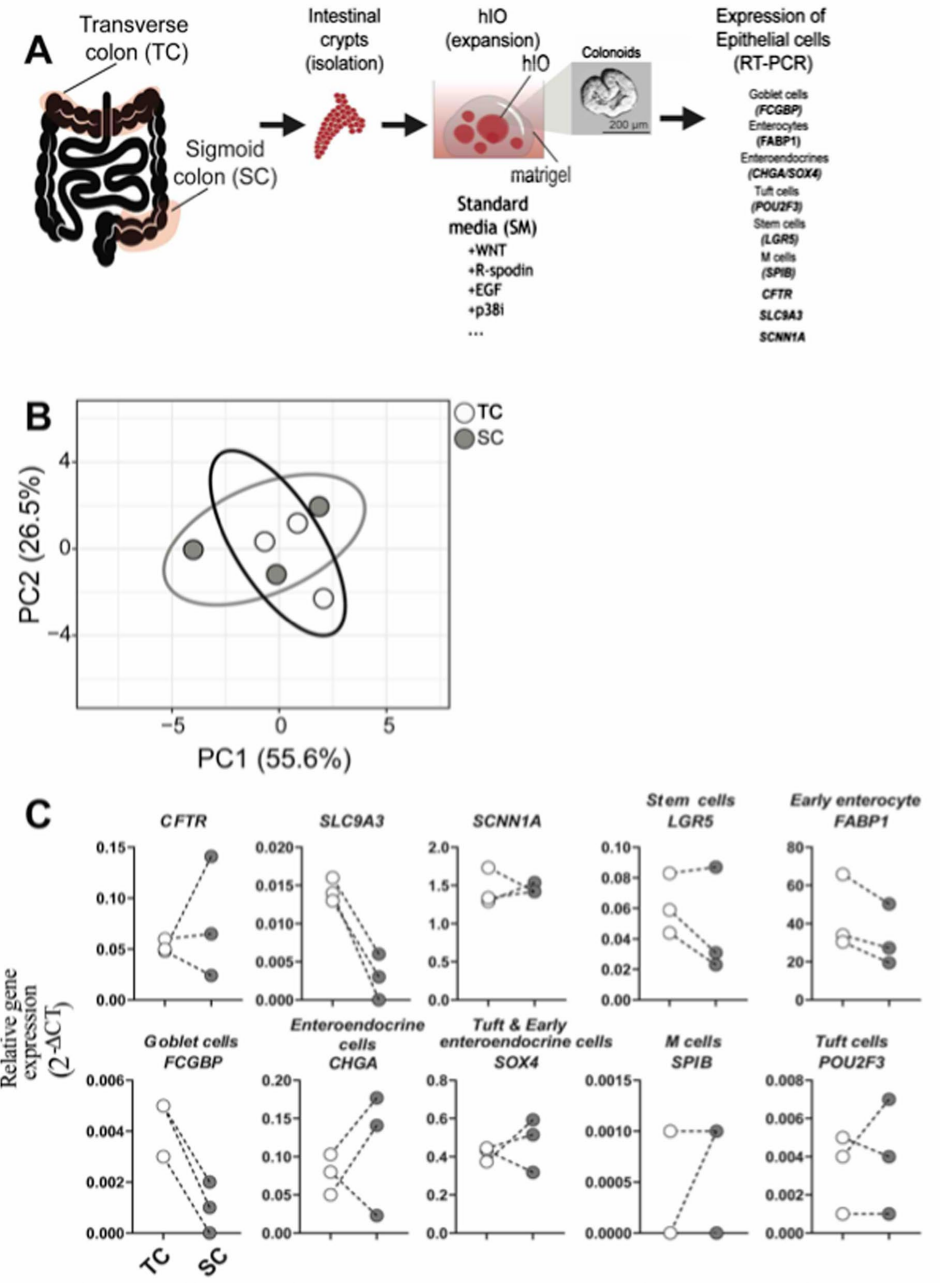
Composition of hIOs derived from transverse and sigmoid colon. hIOs derived from transverse colon (TC) and sigmoid colon (SC) biopsies (n = 3) from non-IBD donors were expanded over 12 days under standard media (SM). Epithelial composition was evaluated on day 12 by RT-qPCR. **A.** Scheme depicting the overall experiment. **B.** Principal component analysis (PCA) from expression of epithelial cells of TC- and SC hIO. **C.** Relative gene expression (2^−ΔCT^) of epithelial surface marks, crypt markers, and epithelial cell marker between TC- and SC hIO

We collected biopsies from both TC and SC of the same volunteers, grew TC- and SC-hIO for 12 days and harvested samples to test the expression of epithelial cell types via RT-PCR of cell-specific markers (Fig. 1A). We analysed crypt maturation via expression of cystic fibrosis transmembrane conductance regulator (*CFTR*), and surface epithelial maturation of TC-derived hIO via expression of sodium–hydrogen exchanger 3 (NHE3; encoded by *SLC9A3* (solute carrier family 9 member A3)) and epithelial sodium channel (ENaC; encoded by *SCNN1A* (sodium channel epithelial 1 subunit alpha)) for SC-derived hIOs (Fig. 1A) [22, 23]. TC- and SC-derived hIO had similar frequencies of epithelial cells, defined by gene expression (Fig. 1B)*.* However, we observed a higher expression of genes representing goblet cells (*FCGBP,* Fc-gamma binding protein) in TC-derived (0.004 ± 0.001) than SC-derived hIOs (0.001 ± 0.001) (Fig. 1C). Goblet cells produce mucin, a key component of intestinal mucus that serves as a barrier for the immune host defence against luminal microbiota but also allows constant nutrient absorption [24]. Fcgbp is the main binding protein of Muc2 [25]. Both *SPIB* (Transcription factor SpiB; M cells) and *POU2F3* (Pit-Oct-Unc (POU) class 2 homeobox 3; Tuft cells) were detected at low expression in both TC- and SC-derived hIOs.

NHE3 (*SLC9A3*) is highly expressed in the surface epithelium of the proximal colon (including TC) where most fluid reabsorption occurs [22, 26]. We observed higher expression in TC-derived hIOs (0.014 ± 0.001) compared to SC-derived hIOs (0.003 ± 0.003; Fig. 1C). ENaC (*SCNN1A*) is normally expressed in the surface epithelium of the distal colon, which includes the SC, and can be used as a marker for epithelium maturation of SC-derived hIOs [23]. However we did not observe a high expression of EnaC (*SCNN1A*) in SC-derived hIOs (Fig. 1C).

TC- and SC-hIOs had similar expression of genes for differentiated epithelial cell types, with the exception of goblet cells. The difference in the transcript levels of the goblet cell marker in SC-hIOs compared to TC-hIOs is similar to the difference seen in the human colon, where SC has lower numbers of sulphated Muc2 + goblet cells compared to TC [27, 28]. We demonstrate that generation of SC-hIOs is feasible.

#### Human sigmoid colon derived intestinal organoids can be generated from frozen biopsies

**Fig. 2:**
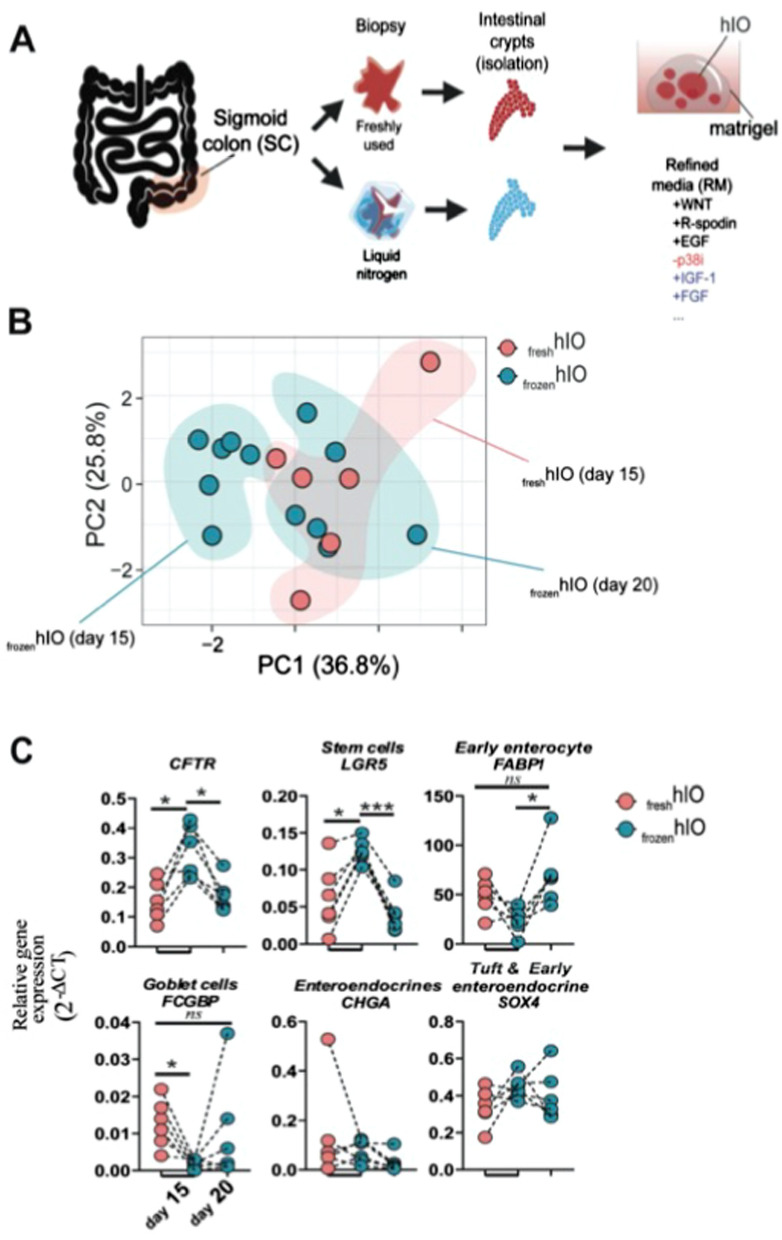
Comparison analysis of epithelial composition between organoids derived from fresh versus frozen biopsies. hIO were grown for 20 d from fresh and frozen SC biopsies (1 month at liquid nitrogen) (n = 6). Epithelial composition was evaluated on days 15 and 20 by RT-qPCR. **A.** Scheme depicting the overall experiment. **B.** PCA from RT-qPCR relative gene expression (2^−ΔCT^) data _fresh_hIO (day 15) and _frozen_hIO (day 15,20). **C.** Relative gene expression between _fresh_hIO (day 15) and _frozen_hIO (day 15,20). ANOVA one-way followed by Sidak’s multiple comparisons test were performed.* p < 0.05, *** p < 0.001, ns = not significant

A drawback of the hIO system is that organoids are usually generated from freshly acquired patient tissue. This practice is limited by the collection of biopsies from local centres with immediate access to research labs. We evaluated the generation of hIOs from frozen biopsies and the impact of cryopreservation on gene expression. We grew SC-hIOs derived from freshly acquired biopsies for 15 days, or SC-hIOs derived from frozen biopsies (cryopreserved for 1 month) from the same donor. After 15 and 20 days of growth, we measured the expression of genes related to cell markers via RT-PCR (Fig. 2A).

_frozen_hIO (day 15) epithelial cell expression differed from that of _fresh_hIO (day 15) and _frozen_hIO (day 20) (Fig. 2B). The _frozen_hIO (day 15) demonstrated high expression of *CFTR* (0.3193 ± 0.08732) compared to _fresh_hIO (day 15) (0.1520 ± 0.06552) and _frozen_hIO (day 20) (0.1698 ± 0.05636) (Fig. 2C). We observed an increase in the stem cell marker, *LGR5* (leucine rich repeat containing G protein-coupled receptor 5), in _frozen_hIO (day 15) (Fig. 2C), whereas expression of genes representing early enterocytes and goblet cells was less in _frozen_hIO (day 15) but fully or partially restored in _frozen_hIO (day 20) (Fig. 2C), indicating the immature status of the organoids. Although _frozen_hIO (day 15) and _fresh_hIO (day 15) differed in respect to expression of cell markers related to epithelial cells, these differences were reduced in _frozen_hIO (day 20), indicating that cryopreservation of SC-derived biopsies likely does not impact generation of hIOs*.* These data demonstrate the feasibility of cryopreserved biopsies in the generation of hIOs, enhancing the potential for nationwide collaboration and cryopreservation of SC-derived biopsies for later clinical use.

#### Addition of IGF-1 and FGF-2 increased expression of epithelial cell markers in human intestinal organoids

**Fig. 3:**
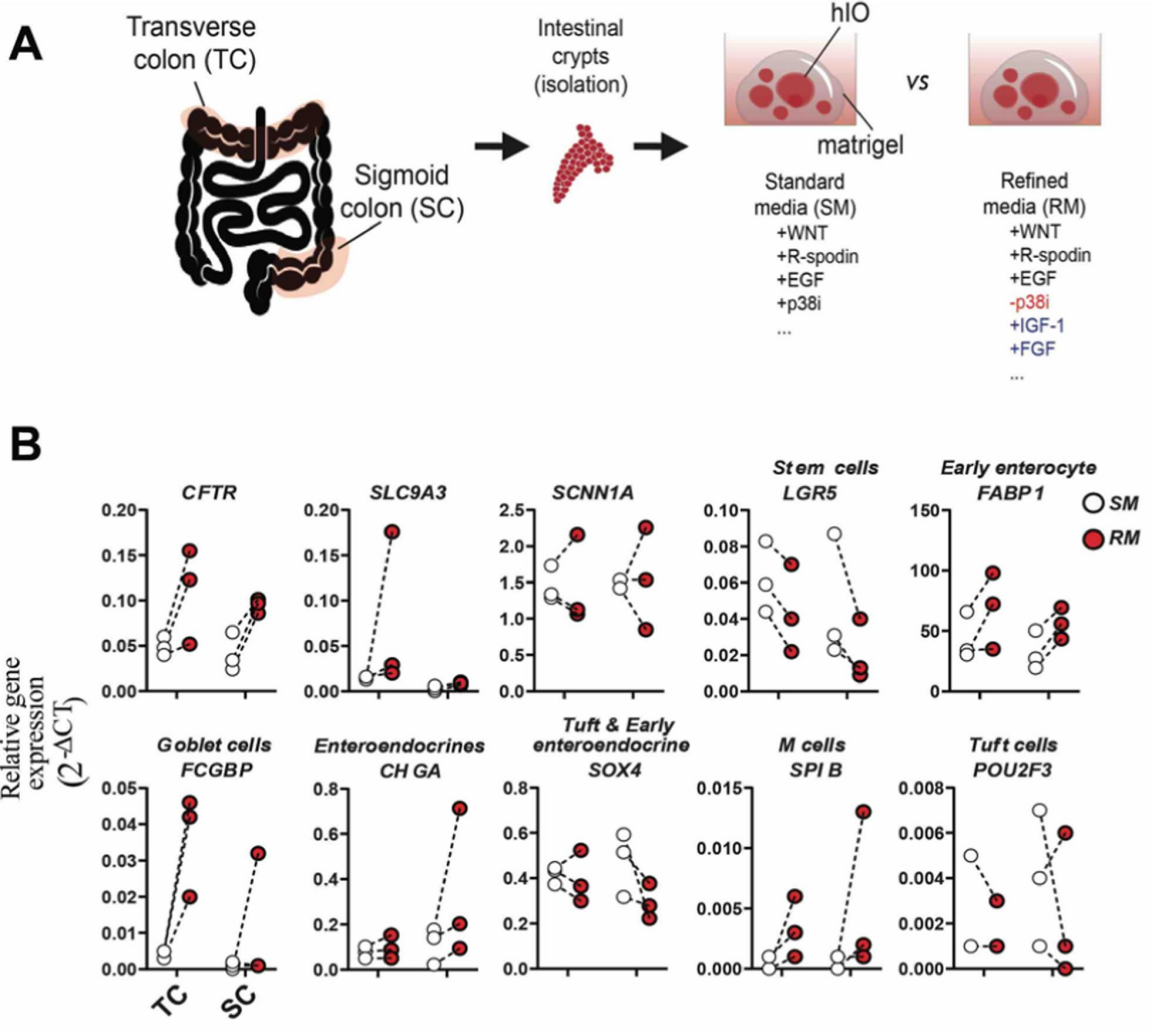
Effect of refined organoid media in the epithelial composition of TC and SC organoids. Healthy organoids derived from TC and SC biopsies (n = 3) were expanded over 12 days with either SM or RM. The epithelial composition of hIO was evaluated by RT-qPCR on day 12. **A.** Scheme depicting the overall experiment. **B.** Relative gene expression (2^−ΔCT^) of epithelial composition of TC- and SC-hIO

While standard organoid media promotes the generation of hIOs, it also reduces the expression of specific intestinal epithelial cells, such as goblet cells [8, 29, 30]. We evaluated the possibility of growing hIO with a refined media (RM; IGF-1, FGF-2, epidermal growth factor (EGF), without p38i; Additional file 1: Data S3) [31]. We compared hIOs generated using RM with those generated using standard media (SM; EGF, p38i) from both TC and SC biopsies (Fig. 3A).

hIOs generated in RM had higher expression of *FCGBP* in TC-hIOs (SM: 0.004 ± 0.001; RM: 0.037 ± 0.014) (Fig. 3B) but not SC-hIOs. Both *SPIB* and *POU2F3* (representing M cells and Tuft cells, respectively) were detected at low expression in TC- and SC-derived hIOs cultured in either SM or RM. hIOs cultured in RM had higher expression of *CFTR* in both TC (SM: 0.004 ± 0.001; RM: 0.037 ± 0.014) and SC (SM: 0.040 ± 0.021; RM: 0.094 ± 0.007) derived hIOs. RM enhanced early enterocyte (*FABP1*, fatty acid binding protein 1) expression in both TC and SC-hIOs (Fig. 3B) compared to SM. RM increased expression of enteroendocrine (*CHGA*, chromogranin A) cells in SC-hIOs, but not in TC-hIOs (Fig. 3B), whereas in TC-hIOs, RM enhanced *SLC9A3* (SM: 0.014 ± 0.001; RM: 0.075 ± 0.087). RM may suppress *LGR5* expression in both TC and SC-hIOs (Fig. 3B). Taken together, despite the low sample size, RM appears superior to SM in terms of the gene expression level of epithelial cells; therefore our data is aligned with that of Fujii et al. [31].

Our data demonstrated the feasibility of cryopreserved biopsies, corroborating earlier findings of Tsai et al. [16]. They observed a delay in the initial growth of organoids from frozen samples compared to organoids derived from fresh biopsies, but the organoids were indistinguishable, even at transcript level [16]. Further, we have identified a time point (d20) where hIOs generated from frozen biopsies reflect similar hIO composition from freshly acquired samples.

Established by Sato et al. [29], hIOs can be expanded in vitro by recreating the stem cell niche through a combination of growth factors, hormones and other molecules, e.g., EGF, WNT, R-spondin-1, and noggin. However, the use of these molecules leads to rapid organoid growth inhibition over time. EGF, for instance, is crucial for organoid growth but the binding of EGF to the EGF receptor (EGFR) leads to activation of p38-MAPK and downregulation of EGFR, implicating p38-signalling pathway in growth inhibition of hIO system over time [4]. Therefore, inhibition of p38 is critical for stabilisation of EGFR and long-term maintenance of hIOs using the conventional media [29]. However, while this trade-off effect enables long-term maintenance of hIO, suppression of p38 reduces the ability of stem cells to differentiate into specific intestinal epithelial cells, such as goblet cells [8, 29]. We validated a new refined media proposed by Fujii et al. [8]. The new media substitutes suppression of p38 by adding IGF-1 and FGF-2. IGF-1 and FGF-2 play an important role in the differentiation of cells and intestinal epithelium regeneration [32, 33]. Both molecules can be used with or without EGF without p38 inhibition (p38i) for effective hIO expansion [8]. We used a non-differentiation media that favours expansion of hIOs and stem cells, characterised by the presence of WNT and R-spondin; for the proper maturation of hIOs for function studies, we recommended the removal of WNT, R-spondin, and EGF [29, 34].

#### Summary

We have demonstrated that hIOs can be grown from easily accessible SC biopsies and from frozen biopsies, and we propose that the methodology and bedside-to-bench pipeline described here provide opportunities for nationwide collaborative research using hIOs to address a variety of research and clinical questions.

### Limitations:

This work was designed to create robust protocols to facilitate standardised clinical research across multiple centres. The research most likely to benefit from these protocols is the study of IBD. hIOs have been used to study epithelial barrier function in IBD [14, 35], bacterial infection [36, 37] and intestinal epithelial inflammasomes [38]; to predict chemotherapy response in colorectal cancer [39, 40] and ovarian cancer [41], and as a drug screening system for cystic fibrosis patients [42]. However, our protocols have been designed using data acquired from healthy individuals, and not those with tumours, inflammation or other potential defects in intestinal permeability.

## Supplementary Information

**Additional file 1: Table S1.** Donors. **Table S2. **PCR primers. **Data S3.** Media.

## Data Availability

The datasets generated and/or analysed during the current study are not publicly available due to ethical regulation constraints but are available from the corresponding author on reasonable request.

## Abbreviations

CHGA: Chromogranin A
DMEM: Dulbecco’s Modified Eagle Medium
EDTA: Ethylenediaminetetraacetic acid
EGF: Epidermal growth factor
ENaC: Epithelial sodium channel
FABP1: Fatty acid binding protein 1
FCS: Fetal calf serum
FGF: Fibroblast growth factor
hIO: Human intestinal organoid
HPRT1: Hypoxanthine phosphoribosyltransferase 1
IBD: Inflammatory bowel diseases
IGF: Insulin-like growth factor
LGR5: Leucine rich repeat containing G protein-coupled receptor 5
NHE3: Sodium hydrogen exchanger 3
PBS: Phosphate buffered saline
POU: Pit-Oct-Unc
POU2F3: POU class 2 homeobox 3
RM: Refined media
SC: Sigmoid colon
SCNN1A: Sodium channel epithelial 1 subunit alpha
SLC9A3: Solute carrier family 9 member A3
SM: Standard media
SpiB: Transcription factor SpiB
TC: Transverse colon
