# Supplementary material for: Assessment of source material for human intestinal organoid culture for research and clinical use
Source: BMC Res Notes. 2022 Feb 10;15:35. doi: 10.1186/s13104-022-05925-4 (PMC8830126; doi:10.1186/s13104-022-05925-4)
Supplement: Supplementary file 1 — Additional file 1: Table S1. Donors. Table S2. PCR primers. Data S3. Media. [file 13104_2022_5925_MOESM1_ESM.docx]

**Table S1.** Donors

| **Age** | **Sex** | **Medication** |
| --- | --- | --- |
| 49 | Male | Paracetamol |
| 67 | Male | Rivaroxaban, Aspirin, Metoprolol, Cilazapril, Metformin, Atorvastatin, Allopurinol, Cetirizine |
| 58 | Female | Nil |
| 67 | Female | Paracetamol, Aspirin, Metoprolol, Cilazapril, Nitrolingual pumpspray |
| 70 | Female | Escitalopram, Celiprolol, Atorvastatin, Mirtazapine, Accupril, Locoid 0.1% Lipocream, One-alpha caps |
| 80 | Female | Paracetamol, Dabigatran, Metoprolol, Cilazapril, Omeprazole. |
| 39 | Female | Nupentin, Amitriptyline HCL, Mebeverine, Topiramate |
| 65 | Male | Methotrexate, Folic Acid, Simvastatin, Omeprazole, Poly-tears, Aqueous Cream, Cetomacrogol 1000, glycerol cream. |
| 70 | Male | None |

**Table S2.** PCR primers

| **Gene** | **Description I** | **Description II** | **Cat. Number^1^** |
| --- | --- | --- | --- |
| *CFTR* | Cystic fibrosis transmembrane conductance regulator | Crypt epithelium maturation | Hs00357011_m1 |
| *CHGA* | Chromogranin A | Enteroendocrine | Hs00900370_m1 |
| *FABP1* | Fatty Acid Binding Protein 1 | Early enterocyte | Hs00155026_m1 |
| *FCGBP* | Fc Fragment of IgG Binding Protein | Goblet cells | Hs00175398_m1 |
| *HPRT1* | Hypoxanthine-guanine phosphoribosyltransferase | Housekeeping gene | 4333768T |
| *LGR5* | Leucine-rich repeat-containing G-protein coupled receptor 5 | Stem cells | Hs00969422_m1 |
| *POU2F3* | POU Class 2 Homeobox 3 | Tuft cells | Hs00205009_m1 |
| *SCNN1A* | Sodium Channel Epithelial 1 Subunit Alpha (ENaC) | Epithelium maturation (SC) | Hs00168906_m1 |
| *SLC9A3* | Solute Carrier Family 9 Member A3 (NHE3) | Epithelium maturation (TC) | Hs00903842_m1 |
| *SOX4* | SRY-Box Transcription Factor 4 | Tuft & Early enteroendocrine | Hs04987498_s1 |
| *SPIB* | Spi-B Transcription Factor | M cells | Hs00162150_m1 |
| *TNF* | Tumour necrosis factor | Cytokine | Hs00174128_m1 |

^1^All from ThermoFisher

**Data S3.** Media

**SM:** Advanced DMEM + F12, N2 (1X, Invitrogen), B27 (1X, Invitrogen), epidermal growth factor (EGF; 0.05 µg/mL, Invitrogen), normocin (0.1 mg/mL), glutamax (1X, Gibco, MA, USA), LY2157299, R-spondin conditional media (10%, Courtesy of G. Butt, University of Otago), Wnt-3A conditional media (50%, Courtesy of G Butt, University of Otago), transforming growth factor (TGF)β inhibitor, 0.5 µM, PeproTech, NJ, USA), noggin (0.1 µg/mL, PeproTech), gastrin (0.001 mg/mL, PeproTech), prostaglandin E2 (PGE2; 0.01 µM, PeproTech) SB-202190 (p38 inhibitor, 0.01 mM, PeproTech), HEPES (0.01 M, cat no. 25245, Serva, Heidelberg, Germany), N-acetyl-L-cysteine (1 mM, Sigma), nicotinamide (10 mM, Sigma), penicillin-streptomycin (1%, Thermo Fisher Scientific).

**RM:** Advanced DMEM + F12, N2, B27, EGF, normocin, glutamax, LY 2157299, noggin, gastrin, PGE2, R-spondin3 conditional media, Wnt-3A conditional media, HEPES, N-acetyl-L-cysteine, nicotinamide, penicillin-streptomycin, insulin-like growth factor (IGF-1; 0.01 µg/mL, PeproTech), fibroblast growth factor (FGF)-basic (FGF-2, 0.05 µg/mL, PeproTech).
